# Towards Automatic Object Detection and Activity Recognition in Indoor Climbing

**DOI:** 10.3390/s24196479

**Published:** 2024-10-08

**Authors:** Hana Vrzáková, Jani Koskinen, Sami Andberg, Ahreum Lee, Mary Jean Amon

**Affiliations:** 1 School of Computing, University of Eastern Finland, FI-80101 Joensuu, Finland; 2 Samsung Electronics, Suwon 16677, Republic of Korea; 3 Department of Informatics, Indiana University Bloomington, Bloomington, IN 47408, USA

**Keywords:** perceptual-motor control, expertise, eye tracking, deep learning, bouldering

## Abstract

Rock climbing has propelled from niche sport to mainstream free-time activity and Olympic sport. Moreover, climbing can be studied as an example of a high-stakes perception-action task. However, understanding what constitutes an expert climber is not simple or straightforward. As a dynamic and high-risk activity, climbing requires a precise interplay between cognition, perception, and precise action execution. While prior research has predominantly focused on the movement aspect of climbing (i.e., skeletal posture and individual limb movements), recent studies have also examined the climber’s visual attention and its links to their performance. To associate the climber’s attention with their actions, however, has traditionally required frame-by-frame manual coding of the recorded eye-tracking videos. To overcome this challenge and automatically contextualize the analysis of eye movements in indoor climbing, we present deep learning-driven (YOLOv5) hold detection that facilitates automatic grasp recognition. To demonstrate the framework, we examined the expert climber’s eye movements and egocentric perspective acquired from eye-tracking glasses (SMI and Tobii Glasses 2). Using the framework, we observed that the expert climber’s grasping duration was positively correlated with total fixation duration (*r* = 0.807) and fixation count (*r* = 0.864); however, it was negatively correlated with the fixation rate (*r* = −0.402) and saccade rate (*r* = −0.344). The findings indicate the moments of cognitive processing and visual search that occurred during decision making and route prospecting. Our work contributes to research on eye–body performance and coordination in high-stakes contexts, and informs the sport science and expands the applications, e.g., in training optimization, injury prevention, and coaching.

## 1. Introduction

Indoor climbing has evolved from a high-risk, niche activity to a mainstream sport accessible to the general population worldwide. With increased numbers of climbing gyms and different climbing variants (i.e., bouldering, lead climbing, speed climbing), climbing has introduced different perceptual-motor challenges that both benefit cognitive skills and physical fitness. To date, sport science has investigated the underpinnings of the climber’s performance and expertise [1]. As a high-risk high-precision activity, climbing requires a highly dynamic and complex interplay between perception, decision making, and action that support proactive planning and precise execution of the climber’s next actions [2,3,4]. Indeed, to an external observer, climbing is challenging to continuously monitor, document, and analyze due to its fast and dynamic character. In this work, we explore how wearable sensors, such as mobile eye tracking, facilitate access to climber’s visual attention as an indicator of effective action planning.

Prior research has examined climbing mainly from body movements and poses using camera systems positioned in front of the climbing wall and route [5,6,7]. To get deeper insights into the climber’s reasoning, head-mounted systems have been utilized in the studies of route reading, planning, and climbing [1,8,9]. The main interest and bottleneck of such studies has been to investigate attention paid to specific areas of interest or bounding boxes (e.g., holds on the wall) or intervals of interest (e.g., moment of grasp) that need to be manually coded in video frames prior to the analysis [10]. Climbing conditions such as fast head movements, sporadic glimpses at hand and foot placement, and partial visual fields make it challenging for both human coders and object-recognition systems to recognize holds and activities in the recorded videos.

In this work, we investigate how mobile eye tracking combined with deep learning object detection enables momentary analysis in climbing performance. Concretely, we present the framework that detects all holds in the climber’s view (object detection) and infers the moment of the climber’s grasp (activity recognition). To demonstrate the framework in the analysis of the climber’s performance, we examine how well the deep learning-based framework detects holds and grasps compared to human coders (RQ1). Using hold detection and action inference, we investigate how the measures of visual attention describe climbing the route (RQ2), and how they are associated with grasping actions (RQ3). Taken together, we pave the way for automatic affordance detection in high-stakes environments.

## 2. Background

In each moment, a climber makes a decision about how to grasp a hold, position their body, or preserve energy for route completion. To foster success in completing the route, belay partners and coaches provide timely feedback on techniques, postures, or alternative routes [11]. Although the concurrent feedback and analysis improve skill acquisition [12,13], it is often limited or inaccessible due to dynamic and complex climbing setups.

With advances in wearable technologies, researchers have employed a variety of sensors to track body movements, visual attention, and physiology to facilitate detailed performance analysis and optimize training for climbers [8,14,15]. For example, inertial measurement units (IMUs) have allowed for route recognition and monitoring [16], trajectory and orientation assessment as an indicator of climbing fluency [17], and performance evaluation [11]. Using external cameras positioned in front of the wall, prior research has automatically detected the climber on the route, their skeletal posture, and movements of individual body parts. For example, Shiro et al. investigated how closely a novice’s movements on the route mimicked an expert’s movements using the EDN network [5]. Similarly, Sasaki et al. employed a neural network and joint detection to automatically characterize the expert’s position of hands and legs during climbing [7]. Ivanova et al. applied CNN RetinaNet with ResNet-50 backbone on recorded videos to automatically detect lowering and infer rope pulling [6]. Further research has examined climbers’ inherent movements [7], differences between indoor and outdoor climbing [18], and overall climbing techniques [19].

Although a large portion of climbing literature focuses on body movement, additional research aims to incorporate understandings of underlying cognitive processes. That is, one needs to acquire and integrate both perceptual and cognitive skills with motoric skills [20,21,22] to support decision making, action planning and execution, and self-regulation, including stress management [23,24]. Specifically, the climber needs to simultaneously perceive affordances based on visual-tactile information, determine an economic movement, and manage ongoing and next actions to sustain energy at the same time [1,9,25]. To understand these complex processes, prior research has investigated how head-mounted cameras can capture and quantify climbers’ visual attention, and how diverse attention strategies indicate climbers’ skills, fluency, and performance [8].

### 2.1. Mobile Eye Tracking in Climbing Performance

Mobile eye tracking is a technological advance that allows for pervasive monitoring of visual attention with unrestricted head and body movements. Equipped with embedded cameras to track the user’s eyes and the user’s field of view, the eye-tracking glasses simultaneously track the position of the pupil and corneal reflections and map their relationship to the scene. The eye tracker captures the locus of foveal attention in form of eye movements, such as fixations (focus stabilization and processing of perceived information) and saccades (automatic or controlled shift of attention’s orientation). Together, the stream of consecutive fixations and saccades forms a scanpath that reveals the spatio-temporal flow of visual attention [1]. In eye-tracking research, fixations and saccades have been intensively studied in numerous cognitive processes and a variety of applications such as expertise, problem-solving in software development, workload, and stress in medical domains [26,27,28,29,30]. In climbing, eye movements play a vital role as well [31].

As a highly dynamic and complex activity, climbing combines many interacting components that cannot be reduced or simplified [23,32,33]. To solve the challenge on the route, the climber relies not only on superior motor skills, but also on efficient eye gaze and cognitive capabilities [23,34]. Together, these complex processes enable the climber to develop an overall strategy and adjust it promptly based on new perceptual cues while sustaining energy and safety [8,35]. Indeed, a climber’s visual attention is a remarkable resource that both selects and informs about the most viable affordances on the wall [1,9,36].

Decision making starts before grasping the first hold. When the climber previews the route from the ground, they need to perceive and select the most optimal maneuvers and critical spots. Indeed, advanced gaze strategies in route preview have been linked to the climber’s improved performance. For example, climbers employed more sophisticated strategies to get the overall gist of the route, thereafter using simpler strategies (look one to two holds ahead) during actual climbing or solving cruxes [20,37].

When on the route, gaze strategies and tactile exploration seamlessly switch between supporting the climber’s current movements and proactive planning of the next moves (i.e., online control) [21,38]. Many factors influence climbing and are reflected in eye movements and the associated metrics. For example, the number of performatory fixations increases during safety foot placement in complex terrains [39] or in anxiety-inducing high traverses [40]. The number of scanpaths increases with the relative duration of route exploration [37]. Seemingly, the number of fixations increases during visual exploration to maximize information when overcoming a challenge. In the opposite direction, the number of fixations and explorations decrease under anxiety [40], but also with route familiarity and training repetition [24,38] and expertise in coaching [41], when eye movements become more task-oriented. Finally, gaze strategies change with expertise. Expert athletes better utilize proactive gaze behaviors to quickly assess subtle task and environmental cues to make informed decisions and efficiently respond [1,34].

To understand the climber’s eye movements on the route, eye movements have been traditionally analyzed with respect to holds as predefined areas of interest (AOIs). Although selecting AOIs in a stable or static scene (e.g., reading text on the screen) is rather straightforward as drawing bounding boxes around objects [27], the same does not apply to dynamic scenes. Specifically in climbing, the climber’s rapid head movements dynamically change the view of the holds due to approach, angle, or motion blur. The climber’s hands and feet cause partial occlusion of the hold, and the lens distortion of the eye-tracker’s scene changes the shape and size of the holds. In addition, other holds from the surrounding routes get into the view as well [10]. Understandably, prior research has relied on the manual coding of the holds, conducted frame-by-frame. Manual coding, however, becomes strenuous with larger datasets and longer climbing sessions. To automate the process of coding, deep learning for object detection and activity recognition holds great potential.

### 2.2. Deep Learning in Performance Analysis of Climbing

Deep learning has achieved remarkable success in numerous computer-vision tasks, and has outperformed traditional methods in object detection and activity recognition across domains [42,43] with a range of academic and commercial applications [44]. Deep learning has been used to recognize both everyday activities, such as picking up objects by hand, as well as more specific actions, e.g., in sports [42,44,45,46]. Alternatively, object detection and activity recognition have been merged into a single model that can simultaneously detect objects in the scene and recognize their activity [47]. The family of YOLO (You Only Look Once) models have came forth in recent years [48,49] for their lightweight, fast, and robust object detection, with applicability in sports for player detection [50,51], athlete tracking [52], and tracking of small objects in ball games [53]. In these contexts, the scene camera is either stable or moving in repetitive patterns (e.g., in basketball), which makes tracking easier. In eye-tracking in the wild, however, the position of the scene camera is changing fast according to its wearer’s attention, which makes predictions considerably harder compared to prior research.

Prior eye-tracking research has also applied machine and deep learning methods in various contexts [54,55,56]. For example, areas of interest have been detected using YOLOv4 [57] and optical flow estimation in mobile eye tracking, situated in students’ laboratory sessions [58]. Wolf et al. applied Mask R-CNN with additional data processing to generate AOIs and assign eye gaze to them [59]. Using the framework, they examined how attention is distributed in an experimental object-handling task. Similarly, Bartz et al. applied the Mask R-CNN model to detect the areas of interest and the ResNET model to classify the cropped area surrounding the eye gaze in video frames taken from the VISUS dataset [60,61]. Similar to our study, Richardson et al. detected holds on the climbing wall using YOLOv5 [62,63] and a head-mounted camera [64]. However, an in-depth climber’s performance analysis was omitted, since the primary objective of the work was sensory-substitution navigation for climbers with vision impairments.

With a few exceptions, previous studies have not applied object detection methods with mobile eye tracking in climbing contexts. While prior research utilized object detection from external cameras positioned in front of the wall [19], only two studies have detected holds on the wall from a climber’s perspective using a GoPro or eye-tracking glasses [64,65]. To bridge the gap between eye-tracking analysis and automated perceptual-motor analysis, we developed a framework that combines deep learning for object and activity recognition that contextualizes the analysis of climber’s eye movements. Our work aims to uncover the complex relationship between the climber’s perception, decision making, and action with respect to environmental affordances.

## 3. Materials and Method

**Figure 1:**
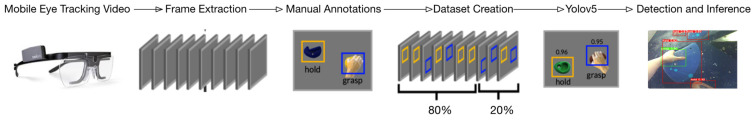
Framework for object detection and activity inference: data collection using eye-tracking glasses, frame extraction, and small-scale manual annotation, and hold and grasp detection (YOLOv5). Tobii 2 glasses (Stockholm, Sweden) image by Tobii AB.

In this work, we present a deep learning-based framework that contributes to research on eye–body performance and coordination in high-stakes contexts. The framework automatically detects all holds in the climber’s view (object detection) and infers the moment of the climber’s grasp (activity recognition), which represents a downstream task. We demonstrate the framework in the analysis of the climber’s visual attention with respect to the inferred activity (summarized in Figure 1).

### 3.1. Case Study in Indoor Climbing

The case study comprised short bouldering sessions with expert climbers. Four high-skilled climbers (male, mean age = 29.25 years, *SD* = 9.53) with extensive climbing experience (number of years climbing: mean = 8.37 years, *SD* = 6.26) volunteered to be recorded during their bouldering practice. Prior to climbing, they were introduced to a mobile eye tracker (Tobii Glasses 2 or SMI Eye-tracking Glasses (Berlin, Germany)). After the three-point eye-tracker calibration, the climbers selected the climbing route and proceeded at their own pace, while their eye movements and field of view were recorded. After the data collection, all videos and eye-tracking outputs were exported using Tobii ProLab 1.194 and SMI BeGaze 3.7.

### 3.2. Data Processing and Small-Scale Manual Coding

**Figure 2:**
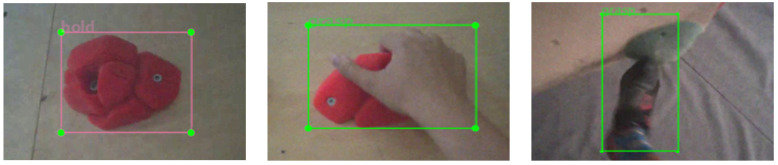
Example object classes: hold (**left**), grasp (**middle**), and foot grasp (**right**). The frames illustrate the characteristics of mobile eye tracking in the climbing context—low image quality, low illumination, narrow view, and distortion—that are typical for mobile eye trackers.

Prior to training the deep learning model, we conducted a randomized, small-scale labeling of selected frames. Egocentric videos were first split into individual frames at 1 frame per second using OpenCV. The small subset of frames (*n* = 320) was first randomly selected from the entire set of frames. The randomized selection of a small sample set was beneficial for fast and efficient prototyping of deep learning-based detection and tuning specifically for this dataset. Next, the objects in the frames were manually coded by drawing bounding boxes tightly around the objects in LabelImg 1.5.0 (https://github.com/HumanSignal/label-studio, accessed on 26 September 2024) and VIA 1.0.7 (https://www.robots.ox.ac.uk/~vgg/software/via/ accessed on 26 September 2024). Figure 2 illustrates manual annotations of hold and grasps. After the coding was completed, the final frameset contained a diverse pool of frames with two classes for object detection, namely the holds on the wall (class: hold) and the hand or foot placed on the hold (class: grasp).

### 3.3. Downstream Task: Object Detection and Activity Recognition in Indoor Climbing

The core of the object detection and activity inference was developed using YOLOv5, the small-scale version of the open-source, object-detection model by Ultralytics LLC (Los Angeles, CA, USA). YOLOv5 is a convolutional neural network that builds upon a backbone network consisting of Cross Stage Partial Networks [66] and a Spatial Pyramid Pooling layer [67], followed by a Path Aggregation Network [68], and a detection head that predicts an object’s location at three different model layers. Three layers of predictions are then combined to predict the bounding boxes for each object. To remove overlapping bounding boxes and multiple detections of the same object, non-maximum suppression is applied to the score of intersection over the union. Finally, the detected object is represented as a set of 2D coordinates of the bounding box, class label, and prediction score (or confidence value) that provides the reliability of detection.

The main advantage of YOLOv5 lies in the combination of data augmentation methods. Apart from standard data augmentation (i.e., rotation, translation, flipping, scaling, and shearing), YOLOv5 also utilizes mosaic data augmentation that concatenates four training images into one mosaic image and crops a new image out of it with the same dimensions as the original training images (introduced in YOLOv4) [225]. Combining all of these aspects together, YOLOv5 offers a tradeoff between fast and robust object detection. Since small-scale studies in niche tasks and domains, such as ours, naturally lack object quantity and diversity suitable for developing a novel deep learning model, fine tuning of pre-trained model represents a viable option. Although climbing holds may not be typically occurring objects, they may share visual features detectable by the pre-trained models, which motivates our study.

**Figure 3:**
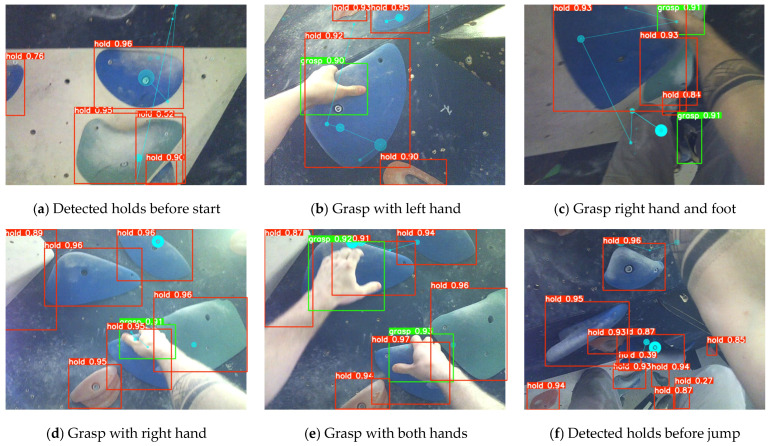
The climber’s view during ascending and before final jump with detected holds (red), grasps (green), and climber’s fixations and saccades (blue). The bounding boxes depict the detected objects (holds; red box) and inferred action (grasp; green box) with the detection confidence.

To prepare collected data for model training, we split the annotated dataset into the training and validation sets in an 80/20 ratio. Next, the detection model was trained for a total of 285 epochs (batch size = 32) to explore possible improvements. When the improvements plateaued, we assessed performance as the weighted average of the mean average precision (mAP) at intersection over union threshold 0.5 and the average mAP for thresholds 0.5–0.95. The best performance was obtained at epoch 88, resulting in a final mAP at 0.5 threshold of 0.8131 and mAP at 0.5–0.95 thresholds of 0.473. The recall was 0.721 and the precision was 0.851. The detection model was applied to all recorded climbing videos, and all detected objects (i.e., individual holds on the wall, and the climber’s grasping of the hold) were extracted in the form of center coordinates and the width and height of the bounding box (as illustrated in Figure 3).

### 3.4. Performance Analysis in Indoor Climbing

To analyze the climber’s eye movements and performance, we computed local and global descriptive metrics of climbing performance. The global metrics were computed from the entire climbing session and comprised the total duration of the climbing, the total number of detected grasps, the total number of fixations, and the total fixation rate. While these metrics were concise when comparing different routes, they cannot pinpoint the local variations in performance. Hence, we examined how eye-tracking data related to detected grasping.

**Figure 4:**
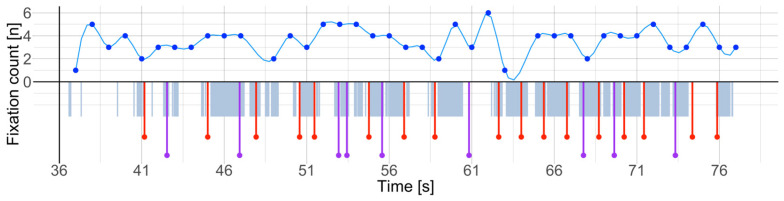
Fixation count during route preview, climbing, and final touch. Fixation count (blue) indicates the moments of increased focus (lower count) and visual exploration (higher count) along with grasps. Automatically detected grasps (grey) are aligned with manually coded grasps (purple) that were visible in eye-tracker’s field of view. The grasps in red were annotated from the previous frames as the climbers grasped the holds without looking at them. Taken together, eye movements and grasps show moments of ascend and immobility and corresponding focus and/or visual exploration.

We segmented the climbing time-series into two activities based on the detected grasps. When a series of consecutive grasps was detected, we denoted the activity as grasping, and the series of non-grasps (when climbers extend their hand or foot from the current hold to the next hold) as reaching. Figure 4 demonstrates the time-series of the segmented grasps and reaches along with the climber’s fixations. In addition, we split the time-series into thirds related to the beginning, middle, and final parts of the climbing (based on the time to completion) and investigated how the performance metrics fluctuated with the perceived difficulty of the route. Grasps below 200 ms long were omitted from the statistical analysis, as they potentially represented spurious classifications. Similarly, grasps without detected fixations and saccades were removed, as they could represented the lack of eye tracker’s fixation and saccade filtering.

For each activity segment, we calculated local metrics, including the climbing segment’s duration and eye movement metrics such as mean and total fixation duration, fixation count, fixation rate, and saccade rate. The eye movement metrics have been closely associated with rapid information processing, selective attention, and visual span [69], which represent critical components of climbing expertise. We leveraged these metrics to examine climbers’ performance. To account for individual differences, all metrics were *z*-score standardized for each climber prior to statistical testing.

## 4. Results

In this work, we investigated how deep learning-driven object detection can facilitate automatic activity recognition and performance analysis in high-stakes environments. First, we assessed the performance of the recognition framework against the ground truth and examined the underpinnings of the detection performance. Next, we applied the detections on recorded eye movements and examined the metrics associated with the detected climbing activity.

### 4.1. RQ1: Automatic and Manual Detection of Holds and Grasps

First, we examined how the deep learning-based framework could detect holds (objects) and grasps (activity) compared to human coders. As a ground truth, climbing routes comprised 13 to 19 holds in total based on the selected route. The framework automatically detected grasping in up to 160 frames (*M* = 40, *SD* = 5.35). Since the number of frames with grasped holds was higher than the number of holds originally placed at the climbing routes, we compared the automatic detections against the ground truth. We manually annotated the moments of the grasps frame-by-frame for all videos (a 40 s climbing video required approximately five minutes of manual coding using Boris [70]), yielding a total of 110 grasps (*M* = 27.5, *SD* = 1.29). Figure 4 illustrates manually annotated grasps against automatically detected grasps on the time-series.

**Figure 5:**
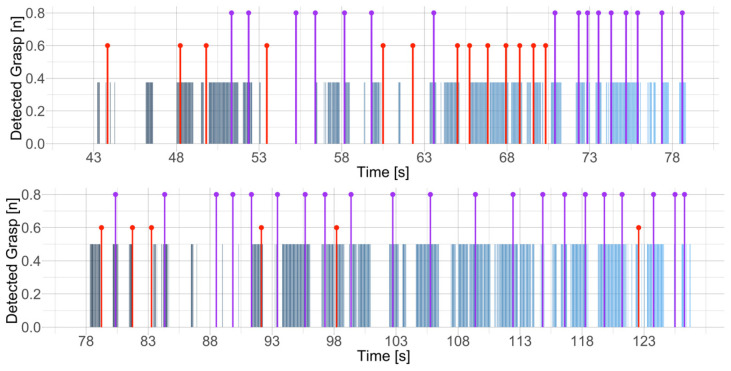
Comparison of automatic grasp detections (blue) and manually coded grasps (purple and red) of two high-skilled climbers. Purple bars denote the grasps that were captured in the video frame, while red bars denote the grasps occurring outside of the scene camera’s field of view. Although grasps were performed out of view, detections captured the grasping hands or feet in the following frames.

When comparing manually and automatically detected grasps, we observed that the high-skilled climbers paid low visual attention to the hold during grasping. They only glanced at the intended hold before grasping and oriented their visual attention to the next move. Because of the narrow angle of the eye tracker’s scene camera, grasping occurred outside the field of view and could not be recorded at the moment of the grasp. In addition, climbers visually visited each hold several times, which was identified as a new grasp and increased the number of recognized grasps. For example, Figure 5 illustrates the time-series of two participants where in the first case, 13 out of 29 grasps occurred out of the field of view. In the latter case, however, only 6 out of 21 grasps occurred out of the field of view.

### 4.2. RQ2: Visual Attention in Climbing Performance

Next, we investigated how the measures of climbing and visual attention characterized the climber’s performance on the route using hold detection and action inference. Since the climbing routes presented different intricacies, the routes were familiar and comparable among participants. The total duration of climbing the route revealed small fluctuations (*M* = 41 s, *SD* = 6) as measured from the first grasp to the final touch in the recorded video. The number of fixations per route ranged between 74 and 131 fixations (*M* = 106, *SD* = 23.90), with an average fixation rate of 2.62 fixations per second (*SD* = 0.65).

**Figure 6:**
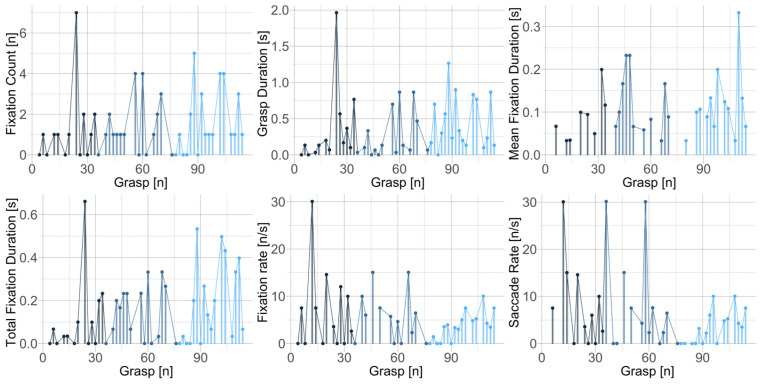
Time series of climbing and eye-tracking metrics of one participant at the beginning (dark blue), middle (blue), and end (light blue) of the climbing. Metrics indicate experienced difficulty, for example, the main crux of the route was presented in the first third, which is apparent in the peak value of grasp duration, fixation count, and total fixation duration.

**Figure 7:**
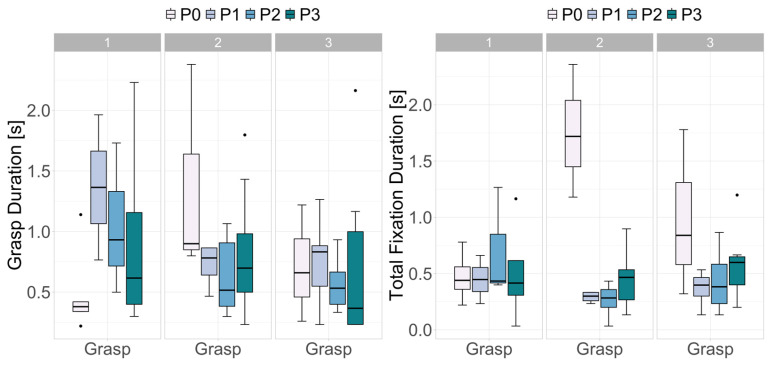
Grasping duration (**left**) and total fixation duration (**right**) of four expert climbers at the start (1), middle (2), and end (3) of the climbing route. While all expert climbers solved the routes approximately at the same pace, their grasping and total fixation durations either decreased or increased over time, suggesting different climbing and visual strategies.

To examine further, we examined how the measures fluctuated during the beginning, middle, and towards the end of climbing. Figure 6 shows the measures on the time-series of one participant. In this case, the first part of the route contained an overhang, which required a specific strategy to overcome the crux and the strength to execute the actions. The climber’s effort was apparent in increased grasp duration, fixation count, and total fixation duration at the beginning of climbing. Similarly, increased difficulty and required strength were apparent in increased durations towards the end of the climb. However, a similar trend was not reported across all the participants. Figure 7 illustrates how grasping duration and total fixation duration of four high-skilled climbers fluctuated with respect to the phase of the climbing and individual characteristics.

### 4.3. RQ3: Grasping in Climbing Performance

Finally, we examined the climbers’ detailed performance with respect to grasping actions and their association with visual attention. Climbers spent on average 0.39 s (*SD* = 0.05) and 0.40 s (*SD* = 0.08) on each grasp and reach. Using repeated measures correlation [71], we associated how the measures of eye movements corresponded to durations of the detected grasps (summarized in Table 1).

Grasping duration was significantly positively correlated with the total fixation duration (*r* = 0.807, *p* < 0.001) and fixation count (*r* = 0.864, *p* < 0.001), which intuitively indicate that more fixations could occur during the longer grasps. However, the positive association could also indicate that during the prolonged grasps (as moments of immobility), climbers could visually explore the next possible moves, resulting in the higher number of fixations. Similarly, during the shorter grasps, their climbing strategy was clear and did not require any further exploration, resulting in the lower number of fixations.

The fixation rate was negatively correlated with the grasping duration (*r* = −0.402, *p* < 0.001), suggesting that the longer grasps were associated with a lower number of longer fixations, indicating deeper cognitive processing, while shorter grasps were accompanied with a higher number of shorter fixations, indicating increased visual search and route prospecting. The fixation rate was also positively correlated with saccade rate (*r* = 0.506, *p* < 0.001). This is an intuitive finding due to the tight relationship between fixations and saccades, although blinks and other eye movements contribute to the bias between the metrics [27].

Interestingly, the mean fixation duration was uncorrelated with the grasping duration (*r* = −0.075, *p* = 0.571). The findings suggest that there were no fast changes in workload associated with the changes in climbing movements, probably due to the fast interplay between movements.

## 5. Discussion

Indoor climbing represents a high-stakes context where dynamic and complex skills act in synergy. Pertinent to our work, grasping has been traditionally investigated in the studies of object affordances where the participants grasped and interacted with an object and task [72]. In climbing, such a task has been to overcome a challenge on the route using not only grasped holds, but with the climber’s entire body. Observing the interplay between body and eye movements has provided novel insights into the climber’s fast decision making and eye–hand coordination. However, the principal challenge of studies with high ecological validity has been in localizing the dynamic spatial areas and temporal intervals of interest that required manual frame-by-frame labeling prior to the analyses. In this work, we have developed a deep learning-based framework for continuous monitoring of holds and grasps in climbing.

For our first research question, we investigated how the deep learning-based framework could detect holds and infer grasps compared to human coders. We observed that while object detection performed well and could detect all visible holds in the video frames with high confidence, grasp inference was less reliable compared to ground truth. Although the detection of grasping achieved high confidence, the occurrences and durations of grasps were too frequent and too short. The main reason for spurious and scattered over-detection was linked to the climber’s visual prospecting and revisiting of the holds on the route, which led to brief and repeated glimpses at the hands and feet. Since these detections did not represent novel grasps and did not correspond to the ground truth, they were filtered out prior to further analyses. To overcome this challenge, future research should focus on automatic methods of object re-identification, as further discussed in the Limitations section.

In the second research questions, we examine how measures of visual attention describe climbing the route. Although significant differences between participants could not be reported with this sample size, our observations were in line with previous research. For example, changes in gaze metrics have been associated with the climber’s learning curve [38] with a mean fixation duration of 252.12 ms (*SD* 8.15), which is comparable to the results we observed (*M* = 223.76 ms, *SD* = 187.35). Our fixation rate results (*M* = 6.56, *SD* = 9.64) also agree with reported fixation rates ranging from 5.71 (*SD* 0.22) to 5.99 (*SD* 0.14) on the regular and irregular route, respectively [3]. The differences received in our results were probably due to the different unit of analysis, our results being based on grasping intervals, whereas results from prior research were received from the entire route.

To address the third research question, we associated the measures of visual attention with grasping. We observed a positive correlation between grasping durations and fixation count and total fixation duration. While the findings were intuitive, they could further indicate the moments of visual exploration during the longer grasps and moments of immobility, and the moments of task-oriented fixations during the fast grasps. These findings are consistent with prior research that climbers employ different visual strategies corresponding to the challenges on the route [1]. Additionally, the time-series of grasping and eye-tracking metrics also revealed fluctuations related to obstacles of strength needed on the route; however, these findings were not significant due to the small sample size and individual characteristics of each climber.

During manual coding, we observed that the climbers paid less attention to the hold at the moment of grasping, with repeated head and gaze shifts before the hand or foot landed on the hold. The asynchronous timing of hand and gaze movements has been related to advanced stages of motor control learning [73]. Anticipatory eye movements and proactive eye gaze (i.e., look-ahead fixations) have been reported for natural and well-practiced tasks [31,74,75]. The ability to focus attention on future targets also depends on factors such as the size of the target, with larger targets requiring less spatial accuracy and consequently less attention [76]. Our findings could be associated with the climber’s proficiency or familiarity with the route.

Application of these findings can increase the training efficiency of novice climbers. Although learning specific visual strategies might be unfeasible, observing how experts solve a specific crux on the wall from their perspective could be insightful [1]. In other domains (e.g., neurosurgery [77]), for example, previous research has shown that when novices were trained to look at relevant locations in the operating field, their learning became more efficient and task-oriented. Furthermore, the automated analysis could illuminate how climbers plan their route, execute the plan, and deviate from it when necessary. Future research could explore automating the risk analysis with focus on the moments of immobility or moments prior to the climber’s fall. Finally, the analysis of eye–hand coordination could complement quantitative and subjective feedback and enrich video-based coaching [78]. Dynamic detection of special areas of interest (holds) and inference of temporal intervals of interests (grasps) in eye-tracking videos is a crucial element that allows us to analyze attention and motor control in contexts where automatic analyses have been impractical or impossible.

### Limitations and Future Research

Despite the use of deep learning frameworks, our work had several limitations induced by the context and climber’s expertise. First, we observed that expert climbers familiar with the route handled the movements in a timely and efficient manner as they looked directly to the grasped hold only during the planning stage and executed the grasp fast without the need for visual confirmation. This was somewhat expected behavior in experts who could rely on brief perceptual cues [1,9,21,79]. While our findings were in line with the literature, our study included only a limited number of climbers at a similar level of expertise, which warranted generalizability of the findings. Future work should increase the sample size to ensure a diversity of climbing strategies and to obtain adequate statistical power to investigate other factors, i.e., skill levels, role of strategy, route difficulty, or personality traits, that influence climbing performance.

For automatic object detection, however, the expert’s behavior presented new challenges. Because grasps occurred outside of the field of view of the eye-tracker’s scene camera, they could not be directly detected. To overcome this challenge, future work could combine the multimodal inference of grasping jointly with bodily and eye-movement patterns and compare the inference to ground truth using a synchronized multicamera setup that combines the climber’s field of view with an external camera positioned in front of the wall [10,38]. Such a setup would enable a deeper understanding of the changing character of constraints in climbing.

Second, lower image quality (imposed by the eye trackers) and strong ceiling illumination of the climbing gym paired with the climber’s handling of movements might have directly impacted detection accuracy. Although these aspects were beyond our control, they inevitably lowered performance detection. Compared to other detection tasks where the external camera is fixed and objects appear or move fairly regularly (e.g., patient’s gait or bodily posture [80,81]), detection of the holds is considerably more challenging. The holds are intentionally manufactured with irregular structure to promote visual and tactile exploration. In addition, expert climbers utilize diverse techniques in how to grasp different types of holds with respect to their affordances and route characteristics. Paired with ceiling illumination and strong shadows, each individual hold can look completely different from a different visual angle, even for the same climber. Consequently, traditional re-identification methods are ineffective since they rely on detecting similar points of interest between frames. To mitigate this issue, future research will need to expand deep learning-based methods for object re-identification and continuous tracking [82,83,84] that incorporate environmental conditions, such as illumination directionality and intensity [85], together with climbing patterns. Taken together, they present the missing piece in robust route tracking from the climber’s perspective.

Finally, related to the environmental conditions, our framework was developed for use in indoor climbing and could not be generalized to the outdoors. While the approach might be transferable to outdoor rock climbing, environmental factors (i.e., weather conditions, illumination changing according to the daytime, appearance of route, i.e., rock and ice waterfalls) present a novel set of challenges that would require extended data collection and low-light enhancement prior to model re-training.

## 6. Conclusions

Sports science has increasingly embraced data science and pervasive sensing, with wearable sensors seamlessly integrating into athletes’ activities to inform on their performance. In climbing, performance on the wall involves not only motor control, but also gaze strategies and cognitive skills. To understand these processes from a climber’s perspective, head-mounted cameras and eye trackers provide access to visual attention, head movements, and limited bodily movement. The laborious analysis of key moments or objects on the route, however, has hindered large-scale adoption and data collection in climbing. To address the key challenges, we have presented a deep learning framework that automatically detects holds and identifies the moments of grasping the hold. We present the first step towards automatic detection of optimal grasp affordances ahead-of-time. These findings create opportunities for application, such as training optimization, coaching, and injury prevention, where gaze strategies reveal the climber’s visual search, decision making, and an immediate action. Future research could expand the intelligent pervasive sensing to demanding tasks such as multimodal detection of perceived stress and difficulty using heart rate and pupillometrics, automatic modeling of learning curves with respect to routes and types of holds, and error prediction in complex climbing scenarios and maneuvers.

## Figures and Tables

**Table 1:** Repeated measures correlation between grasping duration and eye-tracking metrics (numerical values in bold indicate significance *p* < 0.05).

	Grasp Duration	Mean Fixation Duration	Total Fixation Duration	Fixation Count	Fixation Rate	Saccade Rate
**Grasp Duration**		−0.075	**0.807**	**0.864**	**−0.402**	**−0.344**
		(0.571)	**(<0.001)**	**(<0.001)**	**(0.001)**	**(0.007)**
**Mean Fixation Duration**	−0.075		**0.326**	−0.133	−0.158	−0.125
	(0.571)		**(0.011)**	(0.311)	(0.228)	(0.342)
**Total Fixation Duration**	**0.807**	**0.326**		**0.838**	−0.115	−0.213
	**(<0.001)**	**(0.011)**		**(<0.001)**	(0.382)	(0.103)
**Fixation Count**	**0.864**	−0.133	**0.838**		0.008	−0.091
	**(<0.001)**	(0.311)	**(<0.001)**		(0.953)	(0.489)
**Fixation Rate**	**−0.402**	−0.158	−0.115	0.008		**0.506**
	**(0.001)**	(0.228)	(0.382)	(0.953)		**(<0.001)**
**Saccade Rate**	**−0.344**	−0.125	−0.213	−0.091	**0.506**	
	**(0.007)**	(0.342)	(0.103)	(0.489)	**(<0.001)**	

## Data Availability

The datasets presented in this article are not readily available. Due to participants’ privacy and preferences, raw data sharing is not applicable to this article.
